# Withdrawal: "Shelf‐life extension of buffalo meat using Farsi gum edible coating containing Shirazi thyme essential oil", by Elnaz Saffari Samani, Hossein Jooyandeh, Behrooz Alizadeh Behbahani, *Food Science & Nutrition, 2022*

**DOI:** 10.1002/fsn3.2737

**Published:** 2022-01-18

The above article, published online on 18 January 2022 in Wiley Online Library (https://onlinelibrary.wiley.com/doi/full/10.1002/fsn3.2737) in Early View, has been withdrawn by agreement between the Journal’s Editor‐in‐Chief, Prof. Y. Martin Lo, and Wiley Periodicals, LLC. The withdrawal has been agreed because the article was published in error while it was still under peer review. The article has now been rejected and is withdrawn. The authors bear no fault, and we apologize for the error.

Correction added on 24th November 2022, after initial online publication. A duplicate of this article was published under the DOI:10.1002/fsn3.3087. This duplicate has now been deleted and its DOI redirected to this version of the article.

